# Dermatan Sulfate: Structure, Biosynthesis, and Biological Roles

**DOI:** 10.3390/biom15081158

**Published:** 2025-08-12

**Authors:** Congcong Chen, Xuyang Zhang, Weiting Zhang, Dahai Ding, Ravi Sankar Loka, Kun Zhao, Peixue Ling, Shuaishuai Wang

**Affiliations:** 1 National Glycoengineering Research Center, NMPA Key Laboratory for Quality Research and Evaluation of Carbohydrate-Based Medicine, Shandong Key Laboratory of Carbohydrate and Carbohydrate-Conjugate Drugs, Shandong University, Qingdao 266237, China; 2 Suzhou Research Institute of Shandong University, Suzhou 215123, China; 3 Shenzhen Research Institute of Shandong University, Shenzhen 215123, China; 4 Complex Carbohydrate Research Center, University of Georgia, Athens, GA 30602, USA; 5 Department of Medicinal Chemistry, Key Laboratory of Chemical Biology, School of Pharmacy, Cheeloo College of Medicine, Shandong University, Jinan 250012, China

**Keywords:** dermatan sulfate, biosynthesis, preparation, biological functions

## Abstract

Dermatan sulfate (DS) is a remarkably versatile glycosaminoglycan that plays critical roles across a wide array of biological processes. Its unique structure, characterized by repeating disaccharide units of *N*-acetyl-D-galactosamine (GalNAc) and Iduronic acid (IdoA) with variable sulfation patterns, enables it to interact with numerous biomolecules. These interactions mediate diverse functions, including the organization of the extracellular matrix, promotion of wound healing, and modulation of cancer progression. Despite its broad biological relevance, deciphering DS function remains challenging due to its pronounced structural complexity and heterogeneity. Variations in chain length, disaccharide composition, and sulfation patterns make it difficult to fully characterize DS’s intricate structure–function relationships. In this review, recent developments in biosynthesis, preparation, and applications of DS are summarized.

## 1. Introduction

Glycosaminoglycans (GAGs) are a structurally diverse family of complex polysaccharides with repeating disaccharide units. Based on the differences in disaccharide units, GAGs could be classified into four major classes: hyaluronic acid (HA), heparin/heparan sulfate (Hp/HS), chondroitin sulfate/dermatan sulfate (CS/DS), and keratan sulfate (KS) [1]. The structural diversity of GAGs is responsible for their essential roles in numerous biological processes, such as extracellular matrix (ECM) assembly, cell interactions (cell–matrix and cell–cell), cellular signaling, and receptor binding [2].

DS was first isolated from porcine skin by Karl Meyer in the 1940s [3]. Due to its content of hexosamine, an acetyl group, uronic acid, and sulfuric acid in approximately equimolar proportions—similar to those in chondroitin sulfate A—it was initially designated as chondroitin sulfate B [4]. Subsequent studies revealed that DS shares the same *N*-acetyl-D-galactosamine (GalNAc) residue with chondroitin sulfate. However, it differs in the uronic acid component: DS contains L-iduronic acid (IdoA), in contrast to the D-glucuronic acid (GlcA) found in CS [5].

The repeating disaccharide units of DS are linked via alternating β1-4 and α1-3 glycosidic bonds: a β1-4 linkage from GalNAc to IdoA and an α1-3 linkage from IdoA to GalNAc [6]. DS chains are further modified by sulfation at specific positions, the C2 (occasionally C3) of IdoA and the C4 and/or C6 of GalNAc residues. The variable sulfation pattern combined with chain length and the IdoA-to-GlcA ratio differences add to the structural complexity of DS, which further enhances its interactions with functionally relevant proteins.

DS forms DS proteoglycans (DS-PGs) via covalent linkage to core proteins. DS-PGs are widely distributed within ECM and on cell surfaces across various tissues [7,8]. They participate in a wide range of biological processes, such as proliferation, differentiation, migration, adhesion, coagulation, infection, and wound repair [9,10,11,12,13,14]. For example, DS contributes to tissue architecture via ECM assembly and cellular regulation via signaling pathways [15]. Furthermore, mutations in genes involved in the biosynthetic pathway of DS, which impair DS formation, would cause musculocontractural type of Ehlers–Danlos syndrome (mcEDS), a connective tissue disorder disease [16,17]. In contrast, excessive accumulation of DS is a hallmark of several mucopolysaccharidoses (MPS), where deficiencies in DS-degrading enzymes result in intracellular storage [18].

This review summarizes recent advances in DS research, including its biosynthesis, preparation, characterization, and biological functions, with a highlight on skin-related research. Despite the inherent challenges associated with the structural complexity and heterogeneity of DS, advances in analytical techniques are driving deeper insights into its structure–function relationships. These efforts hold promise for uncovering fundamental biological mechanisms and informing therapeutic strategies across a spectrum of human diseases.

## 2. Structure of DS

### 2.1. Primary Structure and Sulfation

**Figure 1:**
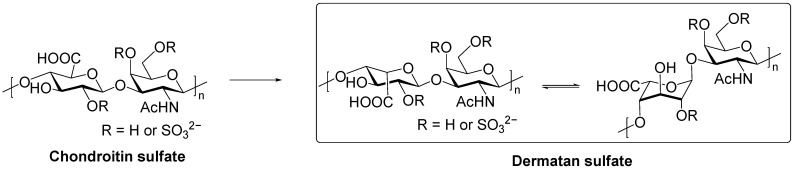
Chemical structure of CS and DS disaccharide unit.

The fundamental structural unit of DS is a repeating disaccharide composed of GalNAc and IdoA (Figure 1). These two monosaccharide units are connected by alternating glycosidic bonds: a β1-4 linked GalNAc and an α1-3 linked IdoA. The IdoA residue can adopt two distinct conformations: a twisted boat (^2^S_0_) and a chair (^1^C_4_) form, occurring at an approximate ratio of 80:20. Among these, the twisted conformation exhibits a more extended geometry [19].

In addition to its glycosidic linkage and monosaccharide composition, the structural diversity of DS is further shaped by differential sulfation. Sulfate groups can be introduced at the C4 and/or C6 positions of the GalNAc residue, and at the C2 position of the IdoA residue (Figure 1). The degree and pattern of sulfation can vary substantially, giving rise to distinct sulfated disaccharide units (Table 1) [20]. For example, DS isolated from swim bladder contains the iA unit (IdoAα1-3GalNAc4S) and the iB unit (IdoA2Sα1-3GalNAc4S) [21]. Additionally, sulfation at the C-3 position of the IdoA residue was also found in nature. Rare 2,3-di-O-sulfated iduronic acid residues were identified in DS extracted from the Pacific starfish *Lysastrosoma anthosticta* [22]. These modifications are believed to be enzymatically regulated and encode biologically meaningful information.

### 2.2. Structural Heterogeneity and Physicochemical Properties

The structure of DS exhibits substantial heterogeneity arising from multiple factors. DS chain length can vary widely, typically comprising between 50 and 200 disaccharide repeats [10]. Additionally, the ratio of IdoA to its C5-epimer GlcA is not fixed and varies depending on tissue origin and physiological context. Moreover, the distribution of IdoA residues along the polysaccharide backbone is variable, further contributing to structural complexity [23].

The physicochemical properties of DS are largely influenced by its structural heterogeneity. The conformational flexibility of IdoA is significantly greater than that of GlcA, leading to decreased chain rigidity in DS relative to CS [24]. This rigidity modulates the conformational dynamics of DS and can influence its interactions with other molecules. Moreover, the presence of multiple sulfate groups imparts a high negative charge density to the DS molecule, rendering DS a highly anionic biopolymer. The charge density and the specific positions of the sulfate groups are critical determinants of DS’s ability to interact with target proteins. Molecular dynamics simulations suggest that increasing the degree of sulfation induces a more rigid and extended conformation of DS in solution [25].

Chain length, uronic acid composition, and sulfation pattern collectively govern the protein-binding capacity and biological activity of DS [24]. This highly regulated structural diversity enables DS to engage with a broad range of proteins with notable specificity—a phenomenon commonly referred to as the “sulfation code” [26].

## 3. Biosynthesis of DS

### 3.1. Biosynthesis of Common Glycosaminoglycan-Protein Linker Region

The biosynthesis of DS is a highly coordinated process that primarily takes place in the Golgi apparatus [27]. It begins with the formation of a conserved linker region composed of the tetrasaccharide GlcAβ1-3Galβ1-3Galβ1-4Xylβ1-O-Ser. This linker region covalently anchors the GAG chain to specific serine residues in the core proteins of proteoglycans.

**Figure 2:**
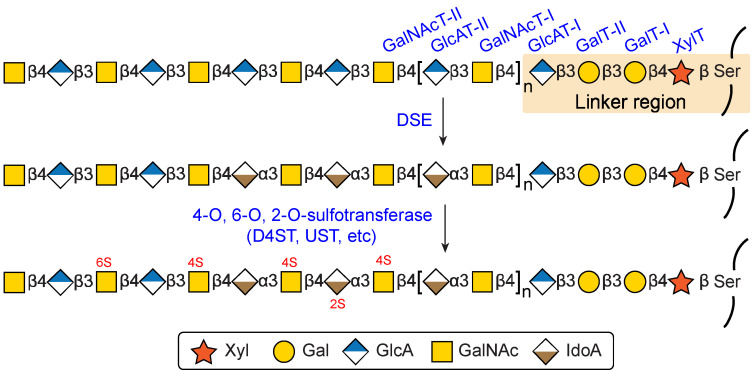
Schematic representation of the DS biosynthetic pathway.

The synthesis of this tetrasaccharide linker is initiated by the transfer of a xylose (Xyl) residue from UDP-Xyl to the serine residue by β-xylosyltransferases (XylT), encoded by the *XYLT1* or *XYLT2* genes [28,29]. This is followed by the sequential addition of two galactose (Gal) residues: the first by β1-4-galactosyltransferase-I (GalT-I), encoded by *B4GALT7* [30], and the second by β1-3-galactosyltransferase-II (GalT-II), encoded by *B3GALT6* [31]. Finally, a GlcA residue is appended by β1-3-glucuronyltransferase-I (GlcAT-I), encoded by *B3GAT3* [32], thereby completing the synthesis of the tetrasaccharide linker (Figure 2).

### 3.2. Biosynthesis of the Repeating Disaccharide Region

Following the formation of the common linker region, the biosynthesis of the CS/DS backbone proceeds via the alternating addition of GlcA and GalNAc residues [33]. This process is initiated by the transfer of GalNAc residue from UDP-GalNAc to the non-reducing end of the tetrasaccharide linker by β1-4-*N*-acetyl-galactosaminyltransferase (GalNAcT-I), encoded by the *CSGALNACT1* or *CSGALNACT2* genes [34].

Upon the formation of the initial pentasaccharide, chondroitin polymerization is catalyzed by CS polymerase complexes, which exhibit both β1-3glucuronyltransferase-II (GlcAT-II) and β1-4-*N*-acetyl-galactosaminyltransferase (GalNAcT-II) activities. These enzymes are encoded by *CHSY1*, *CHSY3*, *CHPF*, and *CHPF2* genes, and together they generate the repeating disaccharide motif of chondroitin (Figure 2) [6]. It should be mentioned that the addition of an α1-4-linked *N*-acetylglucosamine (GlcNAc) residue—rather than a β1-4-linked GalNAc—at the linker region leads to the initiation of heparin/heparan sulfate (Hp/HS) biosynthesis instead [35]. This divergence in sugar selection constitutes a critical branching point in GAG biosynthetic pathways.

### 3.3. Epimerazation and Sulfation

One of the key distinguishing features of DS from CS is the 5-epimerization of GlcA to IdoA [36]. This critical reaction is catalyzed by DS epimerases (DSE), encoded by the *DSE* and *DSEL* genes, respectively [23,37]. These enzymes act on the nascent chondroitin backbone during or after polymer elongation, to generate IdoA-rich domains within the dermatan chain.

Following epimerization, the dermatan backbone undergoes further structural elaboration through site-specific O-sulfation. This process is catalyzed by sulfotransferases that transfer sulfate groups from the sulfate donor 3′-phosphoadenosine 5′-phosphosulfate (PAPS) to specific hydroxyl groups on the sugar residues [38]. Among these, dermatan 4-O-sulfotransferase-1 (D4ST1), encoded by *CHST14* gene, specifically catalyzes sulfation at the C4 position of GalNAc residues adjacent to IdoA. In parallel, uronosyl 2-O-sulfotransferase (UST), encoded by the *UST* gene, catalyzes the sulfation of the C2 position of IdoA. The functional cooperation between DSE1 and D4ST1 is particularly crucial, as it promotes the stabilization and expansion of sulfated IdoA-containing domains. These domains are characteristic of mature DS chains and are central to its ability to engage in protein interactions and fulfill its biological roles [39].

#### 3.3.1. DS Epimerase 1

DS epimerase 1 (DSE1 or DS-epi1), also known as dermatan-sulfate epimerase 1 or chondroitin-glucuronate 5-epimerase, is a key enzyme in the biosynthesis of DS [40]. DSE1 was first purified and functionally characterized from bovine spleen in 2006 [41]. Interestingly, the gene encoding this enzyme had been cloned six years earlier as *SART2* (squamous cell carcinoma antigen recognized by T cells 2) in colorectal carcinoma tissues [42]. At that time, this gene was found to be highly expressed in various tumors, although its function remained unknown.

**Figure 3:**
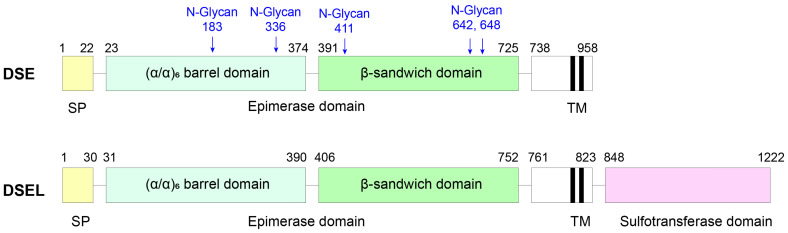
Protein domain structure of DSE1 and DSE2. SP: signal peptides; TM: transmembrane; blue labels indicate the five N-glycosylation sites of DSE1. The structural domains of DSE2 are based on the AlphaFold predication (AF-Q8IZU8-F1).

The full length DSE1 comprises 958 amino acids and contains an N-terminal signal peptide, an epimerase catalysis domain, an uncharacterized region, and a C-terminal trans-membrane domain (Figure 3). In 2016, Tykesson and colleagues demonstrated that a minimum length of 755 is required for its activity. Among several constructs tested, the fragment spanning residues 23–775 yielded the highest expression level, reaching approximately 4 mg/L in HEK293T cells [43].

Their studies also revealed that the shortest active substrate for DSE1 is a tetrasaccharide containing a uronic acid at the reducing end, while octasaccharides or longer oligosaccharides are optimal for efficient catalysis. Notably, the epimerization reaction catalyzed by DSE1 is freely reversible. Since the GlcA configuration is favored over the IdoA configuration at equilibrium, the enzyme tends to retain GlcA residues unless stabilized by downstream modifications. The equilibrium ratio of GlcA to IdoA under uncoupled conditions is approximately 85:15 [44].

In 2021, Hasan et al. reported the first high-resolution crystal structure of DSE1 at 2.4 Å resolution [45]. By integrating macromolecular crystallography with targeted cross-linking mass spectrometry, they constructed a model of the full-length luminal region of the enzyme. This structure consists of an N-terminal (α/α)_6_ barrel domain and a C-terminal β-sandwich domain. Computational docking and molecular dynamics simulations further elucidated the catalytic mechanism, which involves a His/double-Tyr motif. The reaction is initiated by electron delocalization through the carboxylate group of GlcA, followed by proton abstraction at the H5 position mediated by Tyr261, generating a tyrosine anion stabilized by a hydrogen bond from Tyr473. The resulting enol intermediate is further stabilized by His205. Reprotonation at the C5 position is proposed to occur either via a water-mediated proton relay or directly via Asp147. Site-directed mutagenesis confirmed the essential roles of Tyr261, Tyr473, His205, and Asp147 in catalysis. Importantly, structural modeling ruled out protein misfolding as the cause of catalytic inactivity in these mutants.

In addition to its catalytic core, N-glycosylation is critical for enzymatic function of DSE1. Five N-glycosylation sites have been identified (Asn183, Asn336, Asn411, Asn642, and Asn648), with the glycoforms contains high-mannose, complex-type, and core-fucosylated N-glycans. Removal of N-glycans, either through tunicamycin treatment or site-directed mutagenesis, abolishes enzymatic activity, underscoring the structural and functional significance of post-translational glycosylation in DSE1 [45,46].

#### 3.3.2. DS Epimerase 2

Dermatan sulfate epimerase 2 (DSE2 or DS-epi2) is an isozyme of DSE1, sharing a similar enzymatic function but exhibiting distinct expression patterns and domain architecture. While both enzymes are ubiquitously expressed, DSE2 is more prominently expressed in the central nervous system, especially the brain [42,47,48,49]. The *DSEL* gene encoding DSE2 was originally cloned three years prior to its functional annotation during studies on bipolar disorder [47]. The full-length DSE2 protein consists of 1222 amino acids, making it significantly longer than DSE1. While both enzymes share an N-terminal epimerase domain, DSE2 uniquely contains a C-terminal domain similar to that of the heparan/chondroitin 6-O-sulfotransferase family [50]. Despite this predicted similarity, no sulfotransferase activity has been detected either in vitro or in vivo. Overexpression of DSE2 in cell-based systems increases overall epimerase activity but does not alter the sulfation pattern, which remains comparable to that observed in control cells [50].

Thus far, only three classes of known hexuronic acid (HexA) epimerases have been characterized in polysaccharide biology: alginate epimerases, HS epimerases, and DS epimerases [51]. Notably, DSE1 shares no significant sequence similarity with alginate or HS epimerases, nor with any known eukaryotic proteins. Even though both DSE1 and DSE2 catalyze the same epimerization reaction, their overall sequence similarity is surprisingly low: the epimerase domains share only 51% identity, and no sequence similarity is observed between their C-terminal regions. Interestingly, homology has been found between DS epimerases and certain bacterial polysaccharide lyases, especially members of the polysaccharide lyase family 8 (PL8) [45]. Moreover, DSE2 orthologs are present in simpler organisms, whereas DSE1 appears to be restricted to higher eukaryotes. This evolutionary distribution suggests that DSE2 may represent the ancestral form of DS epimerases in humans [45].

#### 3.3.3. Dermatan-4-O-Sulfotransferase 1

Dermatan-4-O-sulfotransferase 1 (D4ST1) is the sixth member of the HNK-1 sulfotransferase family and plays a pivotal role in DS biosynthesis by mediating position-specific sulfation in close coordination with epimerization [52]. D4ST1 catalyzes the transfer of a sulfate group to the 4-hydroxyl position of GalNAc residues, but only when they are adjacent to IdoA units [38]. This strict substrate specificity is functionally important: sulfation of GalNAc residues located adjacent to IdoA, especially near the reducing end of the chain, shifts the epimerization equilibrium catalyzed by DSE toward IdoA formation. Moreover, once GalNAc is sulfated at the C4 position, DSE can no longer revert IdoA back to GlcA, thereby irreversibly locking in the epimerized structure [53].

The tight coordination between epimerization and sulfation is further emphasized by the colocalization of D4ST1 and DSE1 in the Golgi apparatus. Although no direct physical interaction has been demonstrated between the two enzymes, functional enzyme complexes composed of DSE1 and D4ST1 are essential for the formation of long epimerized blocks of 4-O-sulfated DS [39]. In fact, DSE1’s epimerase activity is significantly enhanced in the presence of D4ST1, highlighting the cooperative nature of these enzymes in modulating the structure and biological specificity of DS.

## 4. Preparation and Characterization of DS

DS can be acquired via multiple strategies, including extraction from natural tissues, chemical synthesis, and enzymatic approaches. Among these, tissue extraction remains the most widely used method for large-scale production. In contrast, chemical and enzymatic methods enable the generation of homogenous and structurally defined DS oligosaccharides with precise control over chain length, sulfation patterns, and monosaccharide composition [54]. Recent advances in enzymatic synthesis have further expanded the methodological repertoire for producing tailor-made DS structures [55].

### 4.1. Tissue Extraction

DS is abundant in various animal tissues, including skin, cartilage, intestinal mucosa, and swim bladders. Standard protocols for DS extraction typically involve proteolytic digestion to release GAG chains from core proteins, followed by organic solvent precipitation (e.g., ethanol or cetylpyridinium chloride) and chromatographic purification via ion-exchange or gel filtration techniques [56,57].

In recent years, several innovative extraction strategies have been developed to improve efficiency and sustainability. For instance, an ionic liquid-assisted enzymatic method was reported for the extraction of DS from buffalo hides, which significantly enhanced yield while reducing processing time and environmental impact [58]. Similarly, DS has been successfully purified from bovine collagen waste liquor using sequential filtration and anion-exchange chromatography, eliminating the need for traditional proteolytic digestion and solvent-based steps [59].

The use of non-conventional tissue sources, particularly animal byproducts, has also garnered attention. Fish swim bladders, for example, have been identified as a novel source of DS and DS/CS hybrid chains with notable anticoagulant activity [21]. Additionally, alimentary canals of deer and cattle have been shown to contain high proportions of DS, offering new possibilities for valorizing slaughterhouse waste into high-value biomolecules products [60].

### 4.2. Chemical and Enzymatic Synthesis

While extraction from animal tissues provides bulk DS, chemical synthesis allows the generation of structurally defined DS oligosaccharides with precise control over chain length, disaccharide composition, and sulfation pattern [54]. This approach is especially valuable for investigating the structure–function relationships of specific DS sequences. For example, Jeanneret et al. developed a chemo-selective glycosylation strategy for the iterative synthesis of heparan sulfate- and DS-related oligosaccharides [61]. Such synthetic libraries serve as essential tools for molecular-level mechanistic studies and may support the development of DS-based therapeutic agents. Furthermore, the recent high-throughput preparation of 128 structurally defined heparin hexasaccharides has opened new avenues for the synthesis of well-defined DS analogs [62,63].

In parallel, significant progress has been made in the enzymatic synthesis of other GAGs, including HA [64,65], CS [66,67], HP [68], and KS [69,70]. In 2019, Tykesson et al. established an enzymatic strategy for DS biosynthesis using chondroitin derived from microbial *E. coli* K4 polysaccharide as the backbone [55]. The sequential action of DS biosynthetic enzymes (including DSE1, D4ST1, UST, and GalNAc4,6-sulfotransferase) enabled the generation of DS chains with distinct sulfation patterns. In addition to biosynthetic strategies, chemoenzymatic degradation methods have also been employed to obtain defined DS fragments from native polysaccharides. Linhardt and colleagues utilized chondroitin ABC lyase to prepare DS fragments from polysaccharide chains in a controlled fashion [71,72]. Moreover, enzyme engineering of chondroitin B lyase has improved its catalytic efficiency and specificity, facilitating the production of low-molecular-weight DS for analytical and therapeutic studies [73].

### 4.3. Characterization

DS obtained from natural sources is often present as hybrid DS/CS chains. Therefore, obtaining pure DS and its defined fragments requires the application of advanced purification and characterization techniques [74]. A variety of protocols and workflows have been established for the preparation, separation, and analysis of GAGs, including enzymatic assays for glycosyltransferases and sulfotransferases involved in DS biosynthesis [75,76,77]. It is important to note that standard analytical methods, such as disaccharide composition analysis using chondroitin ABC lyase, cannot effectively differentiate between DS and CS, often leading to misclassification of CS/DS mixtures in the literature [78].

Recent advancements have significantly enhanced the analytical toolbox for DS characterization. Mass spectrometry (MS)-based approaches have emerged as powerful tools. For instance, ion mobility separation (IMS) coupled with MS has been introduced for the screening of HEK293 cells and brain glycosaminoglycomics, offering novel insights into DS composition [79,80]. In addition, mass spectrometry imaging (MSI), when combined with in situ enzymatic digestion and trapped ion mobility spectrometry, enables the spatially resolved profiling of GAGs with detailed sulfation patterns and isomeric structures. This integrated approach facilitates high-resolution mapping of DS distribution across distinct histological regions, offering tissue-specific insights into its structural diversity. [81].

Capillary electrophoresis (CE) has also proven effective in both qualitative and quantitative DS analysis. A recent strategy using Gaussian distribution-based peak fitting enables the simultaneous quantification of DS and CS with high resolution [82]. Additionally, capillary zone electrophoresis (CZE) coupled to electrospray ionization mass spectrometry (ESI-MS) has been successfully applied for detailed structural analysis of CS/DS [83].

Nuclear magnetic resonance (NMR) spectroscopy remains indispensable for elucidating DS fine structure. It has been used to study DS tetrasaccharides and their interactions with DS-binding proteins such as pleiotrophin, offering atomic-level insights into binding specificity [84,85]. Furthermore, NMR studies also reveal conformational flexibility in the glycosidic linkages and multiple puckering states of the IdoA residue, underscoring the dynamic nature of DS in solution [19].

Nanopore is a third generation DNA sequencing technology that utilizes a nano scale protein pore as a biosensor. As negatively charged nucleic acid chains pass through the nanopore, they induce ionic current change, enabling the sequencing of DNA and RNA [86]. Advances in nanopore analysis have expanded its application to glycan detection and sequencing [87]. As GAGs are polyanionic glycans, nanopores have been used to probe GAGs not only for glycan chain length but also monosaccharide compositions and sulfation patterns [88,89,90,91]. For example, different current blockades were observed from DS and CS by using aerolysin nanopore (AeL), demonstrating the potential to discriminate GlcA and IdoA residue [90].

In addition to experimental techniques, computational and enzymatic tools are increasingly contributing to DS characterization. GlycoMaple, a tool for visualization and estimation of glycan structures based on gene expression profiles, enables the analysis of GAG biosynthetic gene expression levels in various human tissues and tumor tissues, providing gene-level insights into the regulation of DS biosynthesis [92]. Additionally, the glycosaminoglycan domain mapping approach (GAGDoMa) provides a straightforward and versatile method for structural domain mapping of complex mixtures of GAGs, particularly those derived from proteoglycans in cultured cell media [93,94]. Moreover, novel enzymatic tools, such as 4-O-endosulfatases, are being used to investigate the structure–function relationships of DS and CS [95,96].

## 5. Biological Functions

Owing to its structural diversity and highly sulfated backbone, DS exerts a broad spectrum of biological roles. It plays pivotal roles in a wide array of biological processes, including ECM organization, cell adhesion and migration, cellular proliferation, neurite outgrowth, tissue repair, and anticoagulant processes [97]. These multifunctional properties are primarily mediated by specific interactions between DS and numerous biomolecules, such as growth factors, cytokines, chemokines, proteases, and ECM proteins [98].

### 5.1. DS in Extracellular Matrix

DS plays a pivotal role in shaping the structural and functional integrity of skin, primarily through its interactions with collagen in the ECM [99]. The presence of varying levels of DS during polymerization distinctly influences collagen I gel architecture, with higher DS levels leading to a more orderly packed fibril structure and lower DS levels leading to disorderly patterned fibrils [100]. In vitro studies have also demonstrated that DS increases fiber radius and matrix pore size. Notably, DS induces the most pronounced changes in these parameters (fiber radius, pore size, and total fibers) when compared with CS and HA [101]. Mechanistically, the presence of IdoA endows DS with greater conformational flexibility than GlcA-based GAGs, thereby enhancing its protein-binding capacity [102]. This increased flexibility facilitates DS binding to collagen, further stabilizing fibrillar networks [103]. In addition, DS influences the function of fibronectin, a high-molecular-weight glycoprotein of the ECM. Suppression of DSE activity of *Xenopus neural* crest cells impairs their adherence to fibronectin, underscoring the essential role of IdoA-containing CS/DS chains in mediating cell adhesion and migration on fibronectin substrates [104].

At the proteoglycan level, DS-PGs, such as decorin and biglycan, closely associate with collagen fibrils to regulate their assembly and organization [105,106]. For example, decorin binds to collagen and forms a ring-mesh-like structure around collagen fibrils, contributing significantly to the stability and integrity of ECM [107]. While DS chain is important for mediating DS-PGs/ligand interactions, the core protein itself plays a critical role in proteins binding [108]. For instance, decorin contains a single DS/CS chain at the N-terminal domain, and a central region composed of 12 leucine-rich repeats (LRRs), among which LRRs 4–6 serve as high-affinity binding sites for type I collagen [109]. Notably, even when skin fibroblasts from patients with Ehlers–Danlos syndrome secreted ∼50% decorin lacking the DS/CS chain, the absence of the GAG chain had limited impact on its collagen binding affinity [110].

The profound impact of DS on skin structure is further evident in genetic conditions affecting its biosynthesis. Mutations in genes encoding enzymes responsible for DS synthesis, such as *DSE* and *CHST14*, cause connective tissue disorders like various types of Ehlers–Danlos syndrome (EDS), particularly musculocontractural Ehlers–Danlos syndrome (mcEDS). These syndromes are often characterized by skin hyperextensibility, joint hypermobility, and tissue fragility, directly highlighting DS’s critical role in maintaining structural integrity [16]. Studies on *Chst14^−/−^* mice, which serve as a model for skin fragility in mcEDS, have shown very low levels of DS disaccharides in the skin and abnormal collagen arrangements in the submucosa of the colon, further underscoring DS’s importance in skin development and strength [111,112].

### 5.2. DS in Wound Healing

The wound healing process is a well-coordinated response to tissue injury that involves both cells and ECM [113]. After a skin injury, healing typically progresses through four stages in order: hemostasis, inflammation, proliferation, and remodeling [114]. During the proliferative phase, collagen production increases significantly, but the fibrils formed are thin and loosely organized. As healing progresses into the remodeling phase, collagen synthesis slows down, while the collagen fibrils undergo maturation, characterized by increased diameter and the formation of cross-links [115].

DS acts as a cofactor for various growth factors, such as fibroblast growth factor-10 (FGF-10) and FGF-2, which are vital for stimulating keratinocyte proliferation and migration—essential steps in re-epithelialization and wound closure [116]. In vitro studies have further confirmed that DS improves the wound healing capacity of human dermal fibroblasts by promoting their proliferation and migration [117]. Additionally, the molecular size of decorin-associated DS changes during the remodeling phase. The molecular size of DS was increased in healing skin by day 15, then returned to baseline levels after day 35 [118].

Given its regenerative capabilities, DS has also found application in novel tissue engineering constructs [119]. For instance, incorporating DS into three-layered skin substitutes enhances their bioactivity by better mimicking the native skin environment, supporting cellular functions, and promoting effective wound healing and regeneration [120].

### 5.3. DS in Anti-Aging and Cosmetic Applications

In skin physiology, DS contributes to hydration due to its high charge density and excellent water-retention capacity [121]. The reduction in total sulfated GAGs levels, including DS, is a hallmark of intrinsic skin aging, leading to dermis thinning and changes in overall skin quality [122]. Functionally, DS can counteract age-related effects by promoting the proliferation and regeneration of dermal fibroblasts and increasing the synthesis of crucial ECM proteins, such as type III collagen and elastin, which are essential for maintaining dermal functionality [117].

GAGs, including DS, are well-recognized skincare targets due to their high endogenous expression in skin, pleiotropic biological activities, and reduced expression in aged skin [123,124]. DS’S ability to promote fibroblast proliferation, stimulate the synthesis of collagen and elastin, and improve skin hydration makes it a valuable ingredient in anti-aging products. Furthermore, its role in wound healing has led to its incorporation into formulations aimed at promoting skin regeneration and repair. Other marine-derived saccharides, such as L-fucose and CS disaccharides, have also been explored for similar anti-aging effects by promoting the proliferation of dermal fibroblasts and dermal papilla cells and increasing ECM molecule production [125]. Topical application of DS offers localized therapeutic benefits while minimizing systemic exposure and associated side effects.

### 5.4. Anticoagulant and Hemostatic Regulation

Among its diverse functions, DS has been most extensively characterized for its role in modulating coagulation. A key mechanism involves its high-affinity interaction with heparin cofactor II (HCII), a serine protease inhibitor that selectively inactivates thrombin. Binding of DS to HCII enhances the inhibition of thrombin by up to 1000-fold, highlighting its potent anticoagulant potential [126]. In vivo, studies using HCII-deficient mice have revealed that vascular DS critically regulates HCII-dependent antithrombotic activity, underscoring its physiological significance. Recombinant forms of DS further enhance HCII-mediated inhibition of thrombin [55]. Notably, DS exhibits minimal interaction with antithrombin III, distinguishing it from heparin and contributing to a more selective anticoagulant profile.

The clinical potential of DS-based anticoagulants is exemplified by sulodexide, a glycosaminoglycan mixture composed of approximately 80% HS and 20% DS. Sulodexide exhibits multiple pharmacological activities, including antithrombotic, anti-inflammatory, and endothelial-protective effects, and has shown therapeutic promise in conditions such as venous thromboembolism, chronic venous insufficiency, peripheral artery disease, and diabetic nephropathy [127]. In parallel, marine-derived sources of DS—such as garfish skin, bones, and heads—are being investigated as sustainable alternatives for anticoagulant applications, with studies evaluating their structural composition and biological activity [128,129,130].

### 5.5. Disease Pathophysiology

Dysregulation of DS biosynthesis or metabolism has been implicated in a range of human diseases, underscoring its essential role in maintaining tissue integrity and homeostasis [131]. Mutations in DS biosynthetic enzymes, alterations in sulfation patterns, or imbalances in DS content can profoundly affect organ development, immune regulation, tumor progression, and neural function [132].

Defective DS biosynthesis has been directly linked to inherited connective tissue disorders, most notably mcEDS [133]. This condition is caused by biallelic pathogenic variants in either *CHST14*, encoding D4ST1, or *DSE/DSEL*, encoding DS epimerases. Such mutations lead to a near-complete loss of DS and a compensatory accumulation of CS, profoundly altering the glycosylation profile of decorin and other DS proteoglycans. The resulting ECM defects manifest as congenital malformations, skin hyperextensibility, joint laxity, and progressive tissue fragility [16,134].

On the other hand, DS accumulation is implicated in lysosomal storage disorders such as mucopolysaccharidosis type I (MPS-I), caused by α-L-iduronidase deficiency, which leads to intracellular accumulation of CS/DS and HS [135]. Recent studies have further highlighted DS-related alterations in complex pathologies such as arthritis and Duchenne muscular dystrophy (DMD), positioning DS and its associated proteoglycans as emerging biomarkers and potential therapeutic targets in these diseases [136].

### 5.6. Role in Cancer

*DSE* gene was initially identified as *SART2* in colorectal carcinoma tissues, suggesting a potential immunogenic role of DS in cancer [42]. Beyond this, DS has been shown to exert complex and context-dependent functions in cancer biology, where it influences tumor cell behavior, modulates tumor microenvironments, and offers unique opportunities for therapeutic intervention.

Alterations in DS biosynthesis or sulfation patterns have been reported across various tumor types. In breast cancer, the central regions of carcinoma tissues exhibit a marked decrease in DS content accompanied by increased CS levels, compared with benign fibroadenomas [92,137]. Similarly, pancreatic tumors exhibit elevated levels of ΔDi-0S, ΔDi-4S, and ΔDi-6S disaccharides, reflecting extensive remodeling of CS/DS composition within the tumor microenvironment [138]. In lung adenocarcinoma, the relative abundance of 4-O-sulfated CS disaccharides and the 6-O-/4-O-sulfation ratio of CS/DS chains show significant variation depending on the underlying genetic mutations [139].

Additionally, DS-PGs such as endocan are increasingly recognized as contributors to tumor progression and angiogenesis [140]. Unlike most proteoglycans that are membrane-bound or embedded within the ECM, endocan is a soluble DS-PGs that regulates cell function through interactions with various proteins, including integrins, growth factors, and signaling molecules [141]. Structurally, endocan contains a core protein with 165 amino acids and a single DS chain covalently connected to serine residue at position 137 [142]. Similarly to other proteoglycans, both core protein and glycan moiety are essential for its biological functions. Removal of the DS chain or point mutations of the core protein disrupts endocan’s ability to promote cellular behavior, such as vascular endothelial growth, vascular sprouting and tumor growth [143].

In functional studies, DS has been shown to directly affect tumor cell viability. Exposure to high concentrations of DS isolated from various sources—including human fascia lata, fibrosis-affected palmar fascia, porcine skin, and intestinal mucosa—induces necroptotic cell death, resulting in a statistically significant decrease in proliferation of luminal breast cancer cells [144,145].

The structural and biochemical properties of DS have also been harnessed for targeted cancer drug delivery. DS-based nanoparticles have been developed as carriers for cancer therapy, with DS-functionalized systems enabling selective internalization via the CD44 receptor. For example, DS/chitosan nanoparticles loaded with anti-inflammatory peptides effectively target colorectal cancer cells through CD44-mediated endocytosis [146]. Similarly, melanoma cells (B16F10) demonstrate high affinity for DS, enabling the design of DS-based nanotherapeutics for melanoma treatment [147]. A representative system incorporates DS as a core scaffold, functionalized with doxorubicin (DOX). This platform facilitates ROS-triggered drug release, enhances tumor-specific uptake, and induces robust immunogenic cell death, ultimately eliciting a strong anti-tumor immune response [148].

### 5.7. DS in Nervous System Development and Function

DS plays a profound role in the central nervous system (CNS), influencing neuronal development, regeneration, and cellular signaling [149]. A comprehensive review has summarized the neurogenic and neuritogenic functions of hybrid CS/DS chains, highlighting their capacity to serve as markers for neural stem cells and to promote their proliferation and differentiation [150]. These hybrid CS/DS chains also contribute to neural circuit formation by capturing and presenting heparin-binding growth factors to stem or neuronal cells. In parallel, another recent review has emphasized the importance of endocan in the pathophysiology of neurological disorders, suggesting that DS-PGs may exert broader regulatory roles within both normal and diseased neural tissues [151].

Beyond these reviews, several primary studies have provided mechanistic insight into DS functions in the central nervous system (CNS). For instance, DS promotes neuronal differentiation and neurite outgrowth in both mouse and human stem cell models. In mouse embryonic stem cells, DS activates extracellular signal-regulated kinase 1/2 and accelerates neurite outgrowth. Similarly, in human neural stem cells, DS promotes both differentiation and migration, suggesting its potential as a novel neurogenic agent [152].

In addition, DS isolated from the marine organism *Phallusia nigra* (PnD2,6S) has demonstrated unique neurotrophic and antioxidant properties. This DS promotes elongation of neurites in murine neuronal cells and protects against oxidative stress-induced neuronal death. These findings suggest that marine-derived DS may serve as a promising bioactive material for neural regeneration and neurodegenerative disease therapy [153,154].

Furthermore, it has been revealed that DS tetrasaccharide is sufficient for inducing autophosphorylation of anaplastic lymphoma kinase (ALK), a receptor tyrosine kinase expressed in developing neurons during the embryonic and postnatal periods. DS oligosaccharides further potentiate this activation. Previous studies have shown that HS and HP can activate ALK ligands [155]. These findings establish GAGs as novel signaling molecules and shed light on the pathophysiological roles of ALK [156].

### 5.8. DS and Signaling Molecules

Emerging evidence suggests that DS participates in specific interactions with various signaling receptors and proteins, such as the receptor for advanced glycation end (RAGE) products, interferon gamma (IFN-γ), and regulated on activation normal T cell expressed and secreted (RANTES), thereby influencing key biological processes.

RAGE is a signal transduction receptor belonging to immunoglobulin superfamily. It was first discovered as a receptor for the products of nonenzymatic glycation and oxidation of proteins and then identified as a major receptor that mediates inflammation in innate immunity [98,157,158]. It has been reported that RAGE binds to DS’s iA units (IdoAα1-3GalNAc4S) to active RAGE-mediated signaling pathways, while it binds more strongly with HS and CS-E disaccharide (GlcAβ1-3GalNAc4S6S), which indicated that both sulfation patterns and disaccharide compositions are critical for GAGs and RAGE interactions [159,160].

Interferon-γ (IFN-γ) is a pro-inflammatory cytokine secreted by activated T cells and natural killer (NK) cells, and it plays a pivotal role in both innate and adaptive immunity, particularly in early host defense against intracellular pathogens [161]. DS has been shown to facilitate the presentation of IFN-γ by mast cells to other immune effector cells, highlighting its potential role in modulating cytokine availability and intercellular communication [162]. Moreover, decorin has been reported to enhance the stability of IFN-γ in vitro and to potentiate IFN-γ-induced activation of STAT1, a key transcription factor in downstream signaling. Notably, DS was capable of significantly augmenting IFN-γ signaling in contrast to the decorin core protein alone [163].

RANTES, also known as CCL5, is a chemokine involved in the activation and migration of leukocytes during inflammatory responses. Its activity is modulated by interactions with GAGs such as HP, HS, CS, and DS [164]. While HP is classically considered the primary GAG ligand for RANTES, both CS and DS also exhibit significant binding affinity. Notably, DS shows higher affinity than CS, likely due to the presence of IdoA residues, which enhance flexibility and charge interactions [165].

### 5.9. Other Biological Roles and Regulatory Functions

DS functions as a critical regulatory molecule in various physiological contexts. For instance, it regulates autoreactive B cell development at the pre-B cell stage by interacting with the IgH µ chain of the pre-B cell receptor [166]. In addition to its immunological role, DS shows promise as an active component in functional foods and supplements for managing metabolic diseases. Oral DS supplementation has been shown to protect against high-fat diet-induced increases in whole-body adiposity and visceral fat mass, highlighting its possible utility in functional foods and dietary supplements for managing metabolic diseases [167]. Moreover, DS derived from marine sources, such as *Lophius litulon*, has also been reported to alleviate allergic symptoms by binding with major royal jelly protein 1, suggesting its promise in the management of hypersensitivity-related conditions [168]. Furthermore, in the field of regenerative medicine, DS also shows promise in cartilage tissue engineering. Its incorporation into bioinks significantly improves the metabolic activity and functionality of embedded cells, effectively inducing a typical cartilaginous gene expression profile [169]. Finally, DS also interacts with M proteins of Group A *Streptococcus* (GAS) to facilitate the adherence of GAS to human HaCaT keratinocytes [170].

## 6. Conclusions and Future Perspectives

DS is a structural distinctive GAG as it shares features with both CS and HP/HS. DS contains the same GalNAc residue as in the CS chain, as it is biosynthetically derived from CS, and it also contains the IdoA residue that is present in HP/HS, which makes it a structurally complex GAG with diverse biological functions. This hybrid nature has historically posed analytical challenges, as DS is often difficult to distinguish from other GAGs, thereby impeding its physiological characterization. Recent advances in enzymology, synthetic methodologies, and analytical techniques have greatly improved our understanding of DS biosynthesis, structural features, and its functional roles in health and disease. Emerging evidence highlights the biological activities of DS, including roles in wound healing, skin aging, anticoagulation, cancer progression, and nervous system development.

Although advances have been made in DS, several critical challenges remain. First, the obtaining of structurally defined DS is still difficult. Owing to its heterogeneity, which includes chain length, disaccharide composition patterns, iduronic acid content, and sulfation motifs, both extraction from natural sources and chemical or enzymatic synthesis strategies pose difficulties in obtaining well-defined DS glycans. Furthermore, the lack of efficient and standardized methods for the characterization of DS also hinders its physiological studies. Comprehensive structural analysis typically requires the integration of multiple analytical techniques, such as chromatography, MS, and NMR. While these approaches are suitable for oligosaccharide analysis, there remains no robust method for the detailed structural characterization of intact, long-chain DS. Most current studies focus primarily on molecular weight and disaccharide composition, whereas critical parameters such as sulfation motifs, IdoA content, and disaccharide connectivity remain underexplored. Given that DS biological activity is closely related to its fine structure, the absence of effective purification and characterization methods continues to impede in-depth structure–function relationship studies. Second, the molecular mechanism and in vivo studies of DS biological function are still poorly investigated. Although many biological functions of DS have been reported, its specific interactions with receptor proteins have not been well characterized by using advanced analytical techniques. In addition, the minimal oligosaccharide fragment that is required for the physiological function of DS has yet to be elucidated. Clarifying the minimal structural motifs necessary for activity would significantly advance mechanistic studies and accelerate the development of DS-based therapeutic agents.

Future challenges include the precise synthesis of defined DS structures, mapping of DS–protein interactomes, and in vivo functional studies. Addressing these questions will be crucial for translating DS biology into clinical innovation and expanding our broader understanding of glycosaminoglycan-mediated regulation.

## Figures and Tables

**Table 1:** The DS disaccharide unit with different sulfation patterns.

Unit	Sequence
iO unit	IdoAα1-3GalNAc
iA unit	IdoAα1-3GalNAc4S
iB unit	IdoA2Sα1-3GalNAc4S
iC unit	IdoAα1-3GalNAc6S
iD unit	IdoA2Sα1-3GalNAc6S
iE unit	IdoAα1-3GalNAc4S6S
iU unit	IdoA2Sα1-3GalNAc
iT unit	IdoA2Sα1-3GalNAc4S6S

## Data Availability

No new data were created or analyzed in this study. Data sharing is not applicable to this article.

## Abbreviations

The following abbreviations are used in this manuscript:

GAGs	Glycosaminoglycans
DS	Dermatan Sulfate
CS	Chondroitin sulfate
HA	hyaluronic acid
HP	Heparan
HS	Heparan sulfate
KS	keratan sulfate
IdoA	L-iduronic acid
GlcA	D-glucuronic acid
GalNAc	N-acetylgalactosamine
GlcNAc	*N*-acetylglucosamine
DSE	Dermatan sulfate epimerase
D4ST	Dermatan 4-O-sulfotransferase
ECM	Extracellular matrix

## References

[1] Volpi N. (2010). Dermatan sulfate: Recent structural and activity data. Carbohydr. Polym..

[2] Perez S., Makshakova O., Angulo J., Bedini E., Bisio A., de Paz J.L., Fadda E., Guerrini M., Hricovini M., Hricovini M. (2023). Glycosaminoglycans: What Remains to Be Deciphered?. JACS Au.

[3] Meyer K., Chaffee E. (1941). The Mucopolysaccharides of Skin. J. Biol. Chem..

[4] Meyer K., Rapport M.M. (1951). The Mucopolysaccharides of the Ground Substance of Connective Tissue. Science.

[5] Trowbridge J.M., Gallo R.L. (2002). Dermatan sulfate: New functions from an old glycosaminoglycan. Glycobiology.

[6] Mikami T., Kitagawa H. (2013). Biosynthesis and function of chondroitin sulfate. BBA—Gen. Subj..

[7] Hayes A.J., Melrose J. (2018). Glycans and glycosaminoglycans in neurobiology: Key regulators of neuronal cell function and fate. Biochem. J..

[8] Mizumoto S., Kosho T., Yamada S., Sugahara K. (2017). Pathophysiological Significance of Dermatan Sulfate Proteoglycans Revealed by Human Genetic Disorders. Pharmaceuticals.

[9] Hayes A., Sugahara K., Farrugia B., Whitelock J.M., Caterson B., Melrose J. (2018). Biodiversity of CS–proteoglycan sulphation motifs: Chemical messenger recognition modules with roles in information transfer, control of cellular behaviour and tissue morphogenesis. Biochem. J..

[10] Mizumoto S., Yamada S. (2022). The Specific Role of Dermatan Sulfate as an Instructive Glycosaminoglycan in Tissue Development. Int. J. Mol. Sci..

[11] Babuty A., Zykwinska A., Samsonov S.A., Candia N., Veinstein C., Pugnière M., Ngo T.H.G., Sinquin C., Muñoz-Garcia J., Colliec-Jouault S. (2025). Anticoagulant Potential of Modified Sulfated Exopolysaccharides from Deep-Sea Bacteria: Toward Non-Animal Heparin Alternatives. Polysaccharides.

[12] Cai J., Sun Z., Yang Q., Xiao W., Deng S. (2025). Dermatan sulfate regulates apoptosis and osteogenic differentiation of hBMSCs through estrogen-like effects. Int. J. Biol. Macromol..

[13] Stachtea X.N., Tykesson E., van Kuppevelt T.H., Feinstein R., Malmström A., Reijmers R.M., Maccarana M. (2015). Dermatan Sulfate-Free Mice Display Embryological Defects and Are Neonatal Lethal Despite Normal Lymphoid and Non-Lymphoid Organogenesis. PLoS ONE.

[14] Yamada S., Sugahara K. (2008). Potential Therapeutic Application of Chondroitin Sulfate/Dermatan Sulfate. Curr. Drug Discov. Technol..

[15] Malmström A., Bartolini B., Thelin M.A., Pacheco B., Maccarana M. (2012). Iduronic Acid in Chondroitin/Dermatan Sulfate:Biosynthesis and Biological Function. J. Histochem. Cytochem..

[16] Kosho T., Mizumoto S., Watanabe T., Yoshizawa T., Miyake N., Yamada S. (2020). Recent Advances in the Pathophysiology of Musculocontractural Ehlers-Danlos Syndrome. Genes.

[17] Syx D., Delbaere S., Bui C., De Clercq A., Larson G., Mizumoto S., Kosho T., Fournel-Gigleux S., Malfait F. (2022). Alterations in glycosaminoglycan biosynthesis associated with the Ehlers-Danlos syndromes. Am. J. Physiol. Cell Physiol..

[18] Arunkumar N., Vu D.C., Khan S., Kobayashi H., Ngoc Can T.B., Oguni T., Watanabe J., Tanaka M., Yamaguchi S., Taketani T. (2021). Diagnosis of Mucopolysaccharidoses and Mucolipidosis by Assaying Multiplex Enzymes and Glycosaminoglycans. Diagnostics.

[19] Silipo A., Zhang Z., Cañada F.J., Molinaro A., Linhardt R.J., Jiménez-Barbero J. (2008). Conformational Analysis of a Dermatan Sulfate-Derived Tetrasaccharide by NMR, Molecular Modeling, and Residual Dipolar Couplings. ChemBioChem.

[20] Wang W., Shi L., Qin Y., Li F. (2020). Research and Application of Chondroitin Sulfate/Dermatan Sulfate-Degrading Enzymes. Front. Cell Dev. Biol..

[21] Yao Y., Tang H., Ma H., Liu Z., Huang J., Yang X., Zhao L., Yuan Q. (2024). Chondroitin Sulfate/Dermatan Sulfate Hybrid Chains from Swim Bladder: Isolation, Structural Analysis, and Anticoagulant Activity. Mar. Drugs.

[22] Ustyuzhanina N.E., Bilan M.I., Dmitrenok A.S., Tsvetkova E.A., Nifantiev N.E., Usov A.I. (2021). Oversulfated dermatan sulfate and heparinoid in the starfish Lysastrosoma anthosticta: Structures and anticoagulant activity. Carbohydr. Polym..

[23] Mizumoto S., Yamada S. (2023). Histories of Dermatan Sulfate Epimerase and Dermatan 4-O-Sulfotransferase from Discovery of Their Enzymes and Genes to Musculocontractural Ehlers-Danlos Syndrome. Genes.

[24] Pągielska M., Samsonov S.A. (2023). Molecular Dynamics-Based Comparative Analysis of Chondroitin and Dermatan Sulfates. Biomolecules.

[25] Roy R., Jonniya N.A., Kar P. (2022). Effect of Sulfation on the Conformational Dynamics of Dermatan Sulfate Glycosaminoglycan: A Gaussian Accelerated Molecular Dynamics Study. J. Phys. Chem. B.

[26] Shanthamurthy C.D., Gimeno A., Leviatan Ben-Arye S., Kumar N.V., Jain P., Padler-Karavani V., Jiménez-Barbero J., Kikkeri R. (2021). Sulfation Code and Conformational Plasticity of l-Iduronic Acid Homo-Oligosaccharides Mimic the Biological Functions of Heparan Sulfate. ACS Chem. Biol..

[27] Pavão M.S.G., Vilela-Silva A.C., Mourão P.A.S. (2006). Biosynthesis of Chondroitin Sulfate: From the Early, Precursor Discoveries to Nowadays, Genetics Approaches. Adv. Pharmacol..

[28] Grebner E.E., Hall C.W., Neufeld E.F. (1966). Glycosylation of serine residues by a uridine diphosphate-xylose: Protein xylosyltransferase from mouse mastocytoma. Arch. Biochem. Biophys..

[29] Grebner E.E., Hall C.W., Neufeld E.F. (1966). Incorporation of D-xylose-C14 into glycoprotein by particles from hen oviduct. Biochem. Biophys. Res. Commun..

[30] Almeida R., Levery S.B., Mandel U., Kresse H., Schwientek T., Bennett E.P., Clausen H. (1999). Cloning and Expression of a Proteoglycan UDP-Galactose:β-Xylose β1,4-Galactosyltransferase I: A seventh member of the human β4-galactosyltransferase gene family. J. Biol. Chem..

[31] Bai X., Zhou D., Brown J.R., Crawford B.E., Hennet T., Esko J.D. (2001). Biosynthesis of the Linkage Region of Glycosaminoglycans: Cloning and activity of galactosyltransferase ii, the sixth member of the β1,3-galactosyltransferase family (β3GalT6). J. Biol. Chem..

[32] Kitagawa H., Tone Y., Tamura J., Neumann K.W., Ogawa T., Oka S., Kawasaki T., Sugahara K. (1998). Molecular Cloning and Expression of Glucuronyltransferase I Involved in the Biosynthesis of the Glycosaminoglycan-Protein Linkage Region of Proteoglycans. J. Biol. Chem..

[33] Silbert J.E., Sugumaran G. (2002). Biosynthesis of Chondroitin/Dermatan Sulfate. IUBMB Life.

[34] Rohrmann K., Niemann R., Buddecke E. (1985). Two N-acetylgalactosaminyltransferase are involved in the biosynthesis of chondroitin sulfate. Eur. J. Biochem..

[35] Sugahara K., Kitagawa H. (2000). Recent advances in the study of the biosynthesis and functions of sulfated glycosaminoglycans. Curr. Opin. Struct. Biol..

[36] Malmström A., Aberg L. (1982). Biosynthesis of dermatan sulphate. Assay and properties of the uronosyl C-5 epimerase. Biochem. J..

[37] Maccarana M., Malmström A., Taniguchi N., Honke K., Fukuda M., Narimatsu H., Yamaguchi Y., Angata T. (2014). Dermatan Sulfate Epimerases (DSE, DSEL). Handbook of Glycosyltransferases and Related Genes.

[38] Mikami T., Mizumoto S., Kago N., Kitagawa H., Sugahara K. (2003). Specificities of Three Distinct Human Chondroitin/Dermatan N-Acetylgalactosamine 4-O-Sulfotransferases Demonstrated Using Partially Desulfated Dermatan Sulfate as an Acceptor: Implication of differential roles in dermatan sulfate biosynthesis. J. Biol. Chem..

[39] Tykesson E., Hassinen A., Zielinska K., Thelin M.A., Frati G., Ellervik U., Westergren-Thorsson G., Malmström A., Kellokumpu S., Maccarana M. (2018). Dermatan sulfate epimerase 1 and dermatan 4-O-sulfotransferase 1 form complexes that generate long epimerized 4-O-sulfated blocks. J. Biol. Chem..

[40] Malmström A., Fransson L.A. (1975). Biosynthesis of dermatan sulfate. I. Formation of L-iduronic acid residues. J. Biol. Chem..

[41] Maccarana M., Olander B., Malmström J., Tiedemann K., Aebersold R., Lindahl U., Li J.-P., Malmström A. (2006). Biosynthesis of Dermatan Sulfate: Chondroitin-glucuronate C5-epimerase is identical to SART2. J. Biol. Chem..

[42] Nakao M., Shichijo S., Imaizumi T., Inoue Y., Matsunaga K., Yamada A., Kikuchi M., Tsuda N., Ohta K., Takamori S. (2000). Identification of a Gene Coding for a New Squamous Cell Carcinoma Antigen Recognized by the CTL. J. Immun..

[43] Tykesson E., Mao Y., Maccarana M., Pu Y., Gao J., Lin C., Zaia J., Westergren-Thorsson G., Ellervik U., Malmström L. (2016). Deciphering the mode of action of the processive polysaccharide modifying enzyme dermatan sulfate epimerase 1 by hydrogen–deuterium exchange mass spectrometry. Chem. Sci..

[44] Malmström A. (1984). Biosynthesis of dermatan sulfate. II. Substrate specificity of the C-5 uronosyl epimerase. J. Biol. Chem..

[45] Hasan M., Khakzad H., Happonen L., Sundin A., Unge J., Mueller U., Malmström J., Westergren-Thorsson G., Malmström L., Ellervik U. (2021). The structure of human dermatan sulfate epimerase 1 emphasizes the importance of C5-epimerization of glucuronic acid in higher organisms. Chem. Sci..

[46] Pacheco B., Maccarana M., Goodlett D.R., Malmström A., Malmström L. (2009). Identification of the Active Site of DS-epimerase 1 and Requirement of N-Glycosylation for Enzyme Function. J. Biol. Chem..

[47] Goossens D., Van Gestel S., Claes S., De Rijk P., Souery D., Massat I., Van den Bossche D., Backhovens H., Mendlewicz J., Van Broeckhoven C. (2003). A novel CpG-associated brain-expressed candidate gene for chromosome 18q-linked bipolar disorder. Mol. Psychiatry.

[48] Bartolini B., Thelin M.A., Rauch U., Feinstein R., Oldberg Å., Malmström A., Maccarana M. (2012). Mouse development is not obviously affected by the absence of dermatan sulfate epimerase 2 in spite of a modified brain dermatan sulfate composition. Glycobiology.

[49] Akatsu C., Mizumoto S., Kaneiwa T., Maccarana M., Malmström A., Yamada S., Sugahara K. (2010). Dermatan sulfate epimerase 2 is the predominant isozyme in the formation of the chondroitin sulfate/dermatan sulfate hybrid structure in postnatal developing mouse brain. Glycobiology.

[50] Pacheco B., Malmström A., Maccarana M. (2009). Two Dermatan Sulfate Epimerases Form Iduronic Acid Domains in Dermatan Sulfate. J. Biol. Chem..

[51] Thelin M.A., Bartolini B., Axelsson J., Gustafsson R., Tykesson E., Pera E., Oldberg Å., Maccarana M., Malmstrom A. (2013). Biological functions of iduronic acid in chondroitin/dermatan sulfate. FEBS J..

[52] Evers M.R., Xia G., Kang H.-G., Schachner M., Baenziger J.U. (2001). Molecular Cloning and Characterization of a Dermatan-specific N-Acetylgalactosamine 4-O-Sulfotransferase. J. Biol. Chem..

[53] Pacheco B., Maccarana M., Malmström A. (2009). Dermatan 4-O-sulfotransferase 1 is pivotal in the formation of iduronic acid blocks in dermatan sulfate. Glycobiology.

[54] Mende M., Bednarek C., Wawryszyn M., Sauter P., Biskup M.B., Schepers U., Bräse S. (2016). Chemical Synthesis of Glycosaminoglycans. Chem. Rev..

[55] Tykesson E., Maccarana M., Thorsson H., Liu J., Malmström A., Ellervik U., Westergren-Thorsson G. (2019). Recombinant dermatan sulfate is a potent activator of heparin cofactor II-dependent inhibition of thrombin. Glycobiology.

[56] Paththuwe Arachchi M.J., Subash A., Bamigbade G.B., Abdin M., Ulla N., Ayyash M. (2025). Fish byproducts as a sustainable source of glycosaminoglycans: Extraction processes, food applications, nutraceutical advancements, and challenges. Trends Food Sci. Technol..

[57] Yuan Q., Shi X., Ma H., Yao Y., Zhang B., Zhao L. (2024). Recent progress in marine chondroitin sulfate, dermatan sulfate, and chondroitin sulfate/dermatan sulfate hybrid chains as potential functional foods and therapeutic agents. Int. J. Biol. Macromol..

[58] Tarannum A., Ballav S., Rao J.R., Fathima N.N. (2023). Extraction of dermatan sulfate using ionic liquid-assisted enzymatic digestion: An efficient approach. Carbohydr. Res..

[59] Osborne S.A., Daniel R.A., Chen W., Stockwell P., Tyrrell K., Desilva K., Seymour R.B. (2016). Extraction, purification and characterisation of dermatan sulphate from bovine collagen waste liquor. Food Bioprod. Process..

[60] Takeda-Okuda N., Yeon S.-J., Matsumi Y., Matsuura Y., Hosaka Y.Z., Tamura J.-I. (2024). Quantitative, compositional, and immunohistochemical analyses of chondroitin sulfate, dermatan sulfate, and hyaluronan in internal organs of deer (*Cervus nippon centralis* and *C. n. yesoensis*) and cattle (*Bos taurus*). Int. J. Biol. Macromol..

[61] Jeanneret R.A., Dalton C.E., Gardiner J.M. (2019). Synthesis of Heparan Sulfate- and Dermatan Sulfate-Related Oligosaccharides via Iterative Chemoselective Glycosylation Exploiting Conformationally Disarmed [2.2.2] l-Iduronic Lactone Thioglycosides. J. Org. Chem..

[62] Wang L., Sorum A.W., Huang B.-S., Kern M.K., Su G., Pawar N., Huang X., Liu J., Pohl N.L.B., Hsieh-Wilson L.C. (2023). Efficient platform for synthesizing comprehensive heparan sulfate oligosaccharide libraries for decoding glycosaminoglycan–protein interactions. Nat. Chem..

[63] Baryal K.N., Ramadan S., Su G., Huo C., Zhao Y., Liu J., Hsieh-Wilson L.C., Huang X. (2023). Synthesis of a systematic 64-membered heparan sulfate tetrasaccharide library. Angew. Chem. Int. Ed..

[64] Li S., Wang S., Fu X., Liu X., Wang P.G., Fang J. (2017). Sequential one-pot multienzyme synthesis of hyaluronan and its derivative. Carbohydr. Polym..

[65] Li S., Wang S., Qu J., Han J., Yang L., Li Y., Li L., Jia Q., Chen C., Ling P. (2023). Programmable one cycle–one disaccharide unit modular synthesis of hyaluronan and chondroitin hybrid glycans. Green Chem..

[66] Li J., Su G., Liu J. (2017). Enzymatic Synthesis of Homogeneous Chondroitin Sulfate Oligosaccharides. Angew. Chem. Int. Ed..

[67] Yang L., Wang S., Chen C., Liu C., Wang X., Li S., Li Y., Ling P., Wang Z., Fang J. (2024). Programmable Enzymatic Toolbox for the Assembly of Fucosylated Chondroitin Derivatives. ACS Catal..

[68] Deng J.-Q., Li Y., Wang Y.-J., Cao Y.-L., Xin S.-Y., Li X.-Y., Xi R.-M., Wang F.-S., Sheng J.-Z. (2024). Biosynthetic production of anticoagulant heparin polysaccharides through metabolic and sulfotransferases engineering strategies. Nat. Commun..

[69] Wu Y., Bosman G.P., Chapla D., Huang C., Moremen K.W., de Vries R.P., Boons G.-J. (2024). A biomimetic synthetic strategy can provide keratan sulfate I and II oligosaccharides with diverse fucosylation and sulfation patterns. J. Am. Chem. Soc..

[70] Wu Y., Bosman G.P., Vos G.M., Uslu E., Chapla D., Huang C., Moremen K.W., Boons G.-J. (2024). Chemoenzymatic synthesis of keratan sulfate oligosaccharides using UDP-Galactose-6-aldehyde to control sulfation at galactosides. Org. Lett..

[71] Yang H.O., Gunay N.S., Toida T., Kuberan B., Yu G., Kim Y.S., Linhardt R.J. (2000). Preparation and structural determination of dermatan sulfate–derived oligosaccharides. Glycobiology.

[72] Dasgupta F., Irene Masada R., Starr C.M., Kuberan B., Yang H.-O., Linhardt R.J. (2000). Chemoenzymatic preparation of dermatan sulfate oligosaccharides as arylsulfatase B and α-L-iduronidase substrates. Glycoconj. J..

[73] Tian M., Xu Y.-Y., Li Y.-N., Yu S., Wang Y.-L., Ma X.-L., Zhang Y.-W. (2024). Engineering of Substrate-Binding Domain to Improve Catalytic Activity of Chondroitin B Lyase with Semi-Rational Design. Curr. Issues Mol. Biol..

[74] Zhang B., Chi L. (2021). Chondroitin Sulfate/Dermatan Sulfate-Protein Interactions and Their Biological Functions in Human Diseases: Implications and Analytical Tools. Front. Cell Dev. Biol..

[75] Nishihara S., Angata K., Aoki-Kinoshita K.F., Hirabayashi J. (2021). Glycoscience Protocols (GlycoPODv2) [Internet].

[76] Cahyadi D.D., Warita K., Takeda-Okuda N., Tamura J.-I., Hosaka Y.Z. (2023). Qualitative and quantitative analyses in sulfated glycosaminoglycans, chondroitin sulfate/dermatan sulfate, during 3 T3-L1 adipocytes differentiation. Anim. Sci. J..

[77] Liu S., Zhang X., Chen Y., Li Y., Liu X. (2024). Study on the interaction between agglutinin and chondroitin sulfate and dermatan sulfate using multiple methods. Int. J. Biol. Macromol..

[78] Zou R., Xu X., Li F. (2025). Classification and characteristics of bacterial glycosaminoglycan lyases, and their therapeutic and experimental applications. J. Cell Sci..

[79] Sarbu M., Seidler D.G., Clemmer D.E., Zamfir A.D. (2024). Introducing Ion Mobility Mass Spectrometry in Brain Glycosaminoglycomics: Application to Chondroitin/Dermatan Sulfate Octasaccharide Domains. J. Am. Soc. Mass Spectrom..

[80] Sarbu M., Ica R., Sharon E., Clemmer D.E., Zamfir A.D. (2022). Identification and Structural Characterization of Novel Chondroitin/Dermatan Sulfate Hexassacharide Domains in Human Decorin by Ion Mobility Tandem Mass Spectrometry. Molecules.

[81] Devlin A., Green F., Takats Z. (2024). Mass Spectrometry Imaging with Trapped Ion Mobility Spectrometry Enables Spatially Resolved Chondroitin, Dermatan, and Hyaluronan Glycosaminoglycan Oligosaccharide Analysis In Situ. Anal. Chem..

[82] Zhou G., Xu Q., Liu X., Wang K. (2024). Quantitative Analysis of Chondroitin Sulfate and Dermatan Sulfate Using Capillary Electrophoresis with Gaussian Distribution-Based Peak Fitting for Improved Peak Resolution. Sep. Sci. Plus.

[83] Zamfir A.D., Neusüß C., Jooß K. (2022). Capillary Zone Electrophoresis–Electrospray Ionization Tandem Mass Spectrometry for Total Analysis of Chondroitin/Dermatan Sulfate Oligosaccharides. Capillary Electrophoresis-Mass Spectrometry: Methods and Protocols.

[84] García-Jiménez M.J., Gil-Caballero S., Maza S., Corzana F., Juárez-Vicente F., Miles J.R., Sakamoto K., Kadomatsu K., García-Domínguez M., de Paz J.L. (2021). Midkine Interaction with Chondroitin Sulfate Model Synthetic Tetrasaccharides and Their Mimetics: The Role of Aromatic Interactions. Chem. Eur. J..

[85] García-Jiménez M.J., Torres-Rico M., de Paz J.L., Nieto P.M. (2022). The Interaction between Chondroitin Sulfate and Dermatan Sulfate Tetrasaccharides and Pleiotrophin. Int. J. Mol. Sci..

[86] Wang Y., Zhao Y., Bollas A., Wang Y., Au K.F. (2021). Nanopore sequencing technology, bioinformatics and applications. Nat. Biotechnol..

[87] Yao G., Ke W., Xia B., Gao Z. (2024). Nanopore-based glycan sequencing: State of the art and future prospects. Chem. Sci..

[88] Xia K., Hagan J.T., Fu L., Sheetz B.S., Bhattacharya S., Zhang F., Dwyer J.R., Linhardt R.J. (2021). Synthetic heparan sulfate standards and machine learning facilitate the development of solid-state nanopore analysis. Proc. Natl. Acad. Sci. USA.

[89] Im J., Lindsay S., Wang X., Zhang P. (2019). Single Molecule Identification and Quantification of Glycosaminoglycans Using Solid-State Nanopores. ACS Nano.

[90] Bayat P., Rambaud C., Priem B., Bourderioux M., Bilong M., Poyer S., Pastoriza-Gallego M., Oukhaled A., Mathé J., Daniel R. (2022). Comprehensive structural assignment of glycosaminoglycan oligo- and polysaccharides by protein nanopore. Nat. Commun..

[91] Rivas F., Zahid O.K., Reesink H.L., Peal B.T., Nixon A.J., DeAngelis P.L., Skardal A., Rahbar E., Hall A.R. (2018). Label-free analysis of physiological hyaluronan size distribution with a solid-state nanopore sensor. Nat. Commun..

[92] Huang Y.-F., Mizumoto S., Fujita M. (2021). Novel Insight Into Glycosaminoglycan Biosynthesis Based on Gene Expression Profiles. Front. Cell Dev. Biol..

[93] Persson A., Nikpour M., Vorontsov E., Nilsson J., Larson G. (2021). Domain Mapping of Chondroitin/Dermatan Sulfate Glycosaminoglycans Enables Structural Characterization of Proteoglycans. Mol. Cell. Proteom..

[94] Persson A., Vorontsov E., Larson G., Nilsson J. (2020). Glycosaminoglycan Domain Mapping of Cellular Chondroitin/Dermatan Sulfates. Sci. Rep..

[95] Wei L., Xu Y., Du M., Fan Y., Zou R., Xu X., Zhang Q., Zhang Y.-Z., Wang W., Li F. (2023). A novel 4-O-endosulfatase with high potential for the structure-function studies of chondroitin sulfate/dermatan sulfate. Carbohydr. Polym..

[96] Wei L., Xu Y., Du M., Fan Y., Zou R., Xu X., Zhang Q., Zhang Y.-Z., Wang W., Li F. (2023). Data on cloning, expression and biochemical characteristics of a chondroitin sulfate/dermatan sulfate 4-O-endosulfatase. Data Brief.

[97] Karamanos N.K., Piperigkou Z., Theocharis A.D., Watanabe H., Franchi M., Baud S., Brézillon S., Götte M., Passi A., Vigetti D. (2018). Proteoglycan Chemical Diversity Drives Multifunctional Cell Regulation and Therapeutics. Chem. Rev..

[98] Mizumoto S., Yamada S., Sugahara K. (2015). Molecular interactions between chondroitin–dermatan sulfate and growth factors/receptors/matrix proteins. Curr. Opin. Struct. Biol..

[99] Miguez P.A., Bash E., Musskopf M.L., Tuin S.A., Rivera-Concepcion A., Chapple I.L.C., Liu J. (2024). Control of tissue homeostasis by the extracellular matrix: Synthetic heparan sulfate as a promising therapeutic for periodontal health and bone regeneration. Periodontology 2000.

[100] Jyothsna K.M., Sarkar P., Jha K.K., A.S. L.K., Raghunathan V., Bhat R. (2021). A biphasic response of polymerized Type 1 collagen architectures to dermatan sulfate. J. Biomed. Mater. Res. A.

[101] Cortes-Medina M., Bushman A.R., Beshay P.E., Adorno J.J., Menyhert M.M., Hildebrand R.M., Agarwal S.S., Avendano A., Friedman A.K., Song J.W. (2024). Chondroitin sulfate, dermatan sulfate, and hyaluronic acid differentially modify the biophysical properties of collagen-based hydrogels. Acta Biomater..

[102] Vallet S.D., Berthollier C., Ricard-Blum S. (2022). The glycosaminoglycan interactome 2.0. Am. J. Physiol. Cell Physiol..

[103] Douglas T., Heinemann S., Mietrach C., Hempel U., Bierbaum S., Scharnweber D., Worch H. (2007). Interactions of Collagen Types I and II with Chondroitin Sulfates A−C and Their Effect on Osteoblast Adhesion. Biomacromolecules.

[104] Gouignard N., Maccarana M., Strate I., von Stedingk K., Malmström A., Pera E.M. (2016). Musculocontractural Ehlers–Danlos syndrome and neurocristopathies: Dermatan sulfate is required for Xenopus neural crest cells to migrate and adhere to fibronectin. Dis. Models Mech..

[105] Lee D.H., Oh J.-H., Chung J.H. (2016). Glycosaminoglycan and proteoglycan in skin aging. J. Dermatol. Sci..

[106] Zhang G., Chen S., Goldoni S., Calder B.W., Simpson H.C., Owens R.T., McQuillan D.J., Young M.F., Iozzo R.V., Birk D.E. (2009). Genetic Evidence for the Coordinated Regulation of Collagen Fibrillogenesis in the Cornea by Decorin and Biglycan. J. Biol. Chem..

[107] Reed C.C., Iozzo R.V. (2002). The role of decorin in collagen fibrillogenesis and skin homeostasis. Glycoconj. J..

[108] Gubbiotti M.A., Vallet S.D., Ricard-Blum S., Iozzo R.V. (2016). Decorin interacting network: A comprehensive analysis of decorin-binding partners and their versatile functions. Matrix Biol..

[109] Svensson L., Heineg D., Oldberg (1995). Decorin-binding Sites for Collagen Type I Are Mainly Located in Leucine-rich Repeats 4-5. J. Biol. Chem..

[110] Rühland C., Schönherr E., Robenek H., Hansen U., Iozzo R.V., Bruckner P., Seidler D.G. (2007). The glycosaminoglycan chain of decorin plays an important role in collagen fibril formation at the early stages of fibrillogenesis. FEBS J..

[111] Hirose T., Mizumoto S., Hashimoto A., Takahashi Y., Yoshizawa T., Nitahara-Kasahara Y., Takahashi N., Nakayama J., Takehana K., Okada T. (2020). Systematic investigation of the skin in Chst14−/− mice: A model for skin fragility in musculocontractural Ehlers–Danlos syndrome caused by CHST14 variants (mcEDS-CHST14). Glycobiology.

[112] Ono F., Takahashi Y., Shimada S., Mizumoto S., Miyata S., Nitahara-Kasahara Y., Yamada S., Okada T., Kosho T., Yoshizawa T. (2025). Carbohydrate sulfotransferase 14 gene deletion induces dermatan sulfate deficiency and affects collagen structure and bowel contraction. PLoS ONE.

[113] Yang F., Bai X., Dai X., Li Y. (2021). The Biological Processes During Wound Healing. Regen. Med..

[114] Sorg H., Sorg C.G.G. (2022). Skin Wound Healing: Of Players, Patterns, and Processes. Eur. Surg. Res..

[115] Almadani Y.H., Vorstenbosch J., Davison P.G., Murphy A.M. (2021). Wound Healing: A Comprehensive Review. Semin. Plast. Surg..

[116] Plichta J.K., Radek K.A. (2012). Sugar-Coating Wound Repair: A Review of FGF-10 and Dermatan Sulfate in Wound Healing and Their Potential Application in Burn Wounds. J. Burn Care Res..

[117] Galvez-Martin P., Martinez-Puig D., Soto-Fernández C., Romero-Rueda J. (2022). In vitroevaluation of anti-aging and regenerative properties of dermatan sulfate for skin care. FASEB J..

[118] Kuwaba K., Kobayashi M., Nomura Y., Irie S., Koyama Y.-I. (2002). Size control of decorin dermatan sulfate during remodeling of collagen fibrils in healing skin. J. Dermatol. Sci..

[119] Soares da Costa D., Reis R.L., Pashkuleva I. (2017). Sulfation of glycosaminoglycans and its implications in human gealth and disorders. Annu. Rev. Biomed. Eng..

[120] Chocarro-Wrona C., Pleguezuelos-Beltrán P., López de Andrés J., Antich C., de Vicente J., Jiménez G., Arias-Santiago S., Gálvez-Martín P., López-Ruiz E., Marchal J.A. (2025). A bioactive three-layered skin substitute based on ECM components effectively promotes skin wound healing and regeneration. Mater. Today Bio.

[121] Wanitphakdeedecha R., Eimpunth S., Manuskiatti W. (2011). The Effects of Mucopolysaccharide Polysulphate on Hydration and Elasticity of Human Skin. Dermatol. Res. Pract..

[122] Oh J.-H., Kim Y.K., Jung J.-Y., Shin J.-E., Kim K.H., Cho K.H., Eun H.C., Chung J.H. (2011). Intrinsic aging- and photoaging-dependent level changes of glycosaminoglycans and their correlation with water content in human skin. J. Dermatol. Sci..

[123] Wang S.T., Hoe N.B., Betts R.J. (2021). Glycosaminoglycans: Sweet as Sugar Targets for Topical Skin Anti-Aging. Clin. Cosmet. Investig. Dermatol..

[124] Zeng Q., Ding D., Loka R.S., Wang S., Ling P. (2024). Recent advances in exploring the properties and applications of hyaluronan. J. Dermatol. Sci. Cosmet. Technol..

[125] Augustyniak A., McMahon H. (2023). Effect of Marine-Derived Saccharides on Human Skin Fibroblasts and Dermal Papilla Cells. Mar. Drugs.

[126] He L., Giri T.K., Vicente C.P., Tollefsen D.M. (2008). Vascular dermatan sulfate regulates the antithrombotic activity of heparin cofactor II. Blood.

[127] Böhm E.W., Buonfiglio F., Korb C.A., Dauth A., Pfeiffer N., Bręborowicz A., Gericke A. (2024). Potential of Sulodexide in the Treatment of Diabetic Retinopathy and Retinal Vein Occlusion. Thromb. Haemost..

[128] Li K., Li R., Liu Y., Li G., Liu S. (2025). Diversity, mechanism and structure-activity relationships of marine anticoagulant-active polysaccharides: A review. Int. J. Biol. Macromol..

[129] Chikha S.B., Bougatef H., Capitani F., Ben Amor I., Maccari F., Gargouri J., Sila A., Volpi N., Bougatef A. (2023). Composition and Anticoagulant Potential of Chondroitin Sulfate and Dermatan Sulfate from Inedible Parts of Garfish (*Belone belone*). Foods.

[130] Valcarcel J., Novoa-Carballal R., Pérez-Martín R.I., Reis R.L., Vázquez J.A. (2017). Glycosaminoglycans from marine sources as therapeutic agents. Biotechnol. Adv..

[131] Mizumoto S., Yamada S. (2021). Congenital Disorders of Deficiency in Glycosaminoglycan Biosynthesis. Front. Genet..

[132] Mizumoto S., Yamada S. (2021). An Overview of in vivo Functions of Chondroitin Sulfate and Dermatan Sulfate Revealed by Their Deficient Mice. Front. Cell Dev. Biol..

[133] Yoshizawa T., Kosho T. (2023). Mouse Models of Musculocontractural Ehlers-Danlos Syndrome. Genes.

[134] Müller T., Mizumoto S., Suresh I., Komatsu Y., Vodopiutz J., Dundar M., Straub V., Lingenhel A., Melmer A., Lechner S. (2013). Loss of dermatan sulfate epimerase (DSE) function results in musculocontractural Ehlers–Danlos syndrome. Hum. Mol. Genet..

[135] Kubaski F., de Oliveira Poswar F., Michelin-Tirelli K., Matte U.D.S., Horovitz D.D., Barth A.L., Baldo G., Vairo F., Giugliani R. (2020). Mucopolysaccharidosis Type I. Diagnostics.

[136] Maciej-Hulme M.L., Melrose J., Farrugia B.L. (2023). Arthritis and Duchenne muscular dystrophy: The role of chondroitin sulfate and its associated proteoglycans in disease pathology and as a diagnostic marker. Am. J. Physiol. Cell Physiol..

[137] Olsen E.B., Trier K., Eldov K., Ammitzbøll T. (1988). Glycosaminoglycans in human breast cancer. Acta Obstet. Gynecol. Scand..

[138] Ren Q., Wang J., Liu C., Meng L.-X., Qian R.-K., Gao H.-J., Qin W., Zhou C.-J., Qiao S., Wang H.-Y. (2021). Exploring the sulfate patterns of chondroitin sulfate/dermatan sulfate and keratan sulfate in human pancreatic cancer. J. Pharm. Biomed. Anal..

[139] Pál D., Bugyi F., Virág D., Szabó D., Schlosser G., Kovalszky I., Harkó T., Moldvay J., Turiák L. (2025). Analysis and characterization of chondroitin/dermatan sulfate composition of lung adenocarcinoma tissues with different types of genetic alterations in ALK, EGFR and KRAS oncogenes. Carbohydr. Polym. Technol. Appl..

[140] Entezarian M., Ameli F., Masir N., Geok Chin T. (2022). Significance of Endocan Expression in Various Types of Epithelial Ovarian Tumors. Iran. J. Pathol..

[141] Bastola S., Pavlyukov M.S., Sharma N., Ghochani Y., Nakano M.A., Muthukrishnan S.D., Yu S.Y., Kim M.S., Sohrabi A., Biscola N.P. (2025). Endothelial-secreted Endocan activates PDGFRA and regulates vascularity and spatial phenotype in glioblastoma. Nat. Commun..

[142] Yang J., Yang Q., Yu S., Zhang X. (2015). Endocan: A new marker for cancer and a target for cancer therapy (Review). Biomed. Rep..

[143] Pan K.-F., Yang Y.-C., Lee W.-J., Hua K.-T., Chien M.-H. (2022). Proteoglycan Endocan: A multifaceted therapeutic target in Cancer. Biochim. Biophys. Acta (BBA) Rev. Cancer.

[144] Wisowski G., Pudełko A., Olczyk K., Paul-Samojedny M., Koźma E.M. (2022). Dermatan Sulfate Affects Breast Cancer Cell Function via the Induction of Necroptosis. Cells.

[145] Wisowski G., Pudełko A., Paul-Samojedny M., Komosińska-Vassev K., Koźma E.M. (2024). Dermatan Sulfate Affects the Activation of the Necroptotic Effector MLKL in Breast Cancer Cell Lines via the NFκB Pathway and Rac-Mediated Oxidative Stress. Biomolecules.

[146] Blachman A., Birocco A.M., Curcio S., Camperi S.A., Gianvincenzo P.D., Rodriguez J.A., Barredo-Vacchelli G.R., Cenci G., Sosnik A., Moya S. (2023). Dermatan Sulfate/Chitosan Nanoparticles Loaded with an Anti-Inflammatory Peptide Increase the Response of Human Colorectal Cancer Cells to 5-Fluorouracil. Macromol. Biosci..

[147] Li S., Zhang F., Yu Y., Zhang Q. (2020). A dermatan sulfate-functionalized biomimetic nanocarrier for melanoma targeted chemotherapy. Carbohydr. Polym..

[148] Zhang Q., Li S., Ren J., He X., Shi H., Zhang F., Li H., Tong R. (2022). ROS-triggered nanoinducer based on dermatan sulfate enhances immunogenic cell death in melanoma. J. Control. Release.

[149] Hayes A.J., Melrose J. (2021). Neural Tissue Homeostasis and Repair Is Regulated via CS and DS Proteoglycan Motifs. Front. Cell Dev. Biol..

[150] Sugahara K., Mikami T. (2007). Chondroitin/dermatan sulfate in the central nervous system. Curr. Opin. Struct. Biol..

[151] Liu S., Bai T., Feng J. (2024). Endocan, a novel glycoprotein with multiple biological activities, may play important roles in neurological diseases. Front. Aging Neurosci..

[152] Ogura C., Hirano K., Mizumoto S., Yamada S., Nishihara S. (2020). Dermatan sulphate promotes neuronal differentiation in mouse and human stem cells. J. Biochem..

[153] Medeiros T.B., Cosendey P., Gerin D.R., de Sousa G.F., Portal T.M., Monteiro-de-Barros C. (2023). The effect of the sulfation patterns of dermatan and chondroitin sulfate from vertebrates and ascidians on their neuritogenic and neuroprotective properties. Int. J. Biol. Macromol..

[154] de Sousa G.F., Palmero C.Y., de Souza-Menezes J., Araujo A.K., Guimarães A.G., de Barros C.M. (2020). Dermatan sulfate obtained from the Phallusia nigra marine organism is responsible for antioxidant activity and neuroprotection in the neuroblastoma-2A cell lineage. Int. J. Biol. Macromol..

[155] Murray P.B., Lax I., Reshetnyak A., Ligon G.F., Lillquist J.S., Natoli E.J., Shi X., Folta-Stogniew E., Gunel M., Alvarado D. (2015). Heparin is an activating ligand of the orphan receptor tyrosine kinase ALK. Sci. Signal..

[156] Machino M., Gong Y., Ozaki T., Suzuki Y., Watanabe E., Imagama S., Kadomatsu K., Sakamoto K. (2021). Dermatan sulphate is an activating ligand of anaplastic lymphoma kinase. J. Biochem..

[157] Ramasamy R., Yan S.F., Herold K., Clynes R., Schmidt A.M. (2008). Receptor for Advanced Glycation End Products. Ann. N. Y. Acad. Sci..

[158] Mizumoto S., Sugahara K. (2013). Glycosaminoglycans are functional ligands for receptor for advanced glycation end-products in tumors. FEBS J..

[159] Xu D., Young J.H., Krahn J.M., Song D., Corbett K.D., Chazin W.J., Pedersen L.C., Esko J.D. (2013). Stable RAGE-heparan sulfate complexes are essential for signal transduction. ACS Chem. Biol..

[160] Mizumoto S., Takahashi J., Sugahara K. (2012). Receptor for Advanced Glycation End Products (RAGE) Functions as Receptor for Specific Sulfated Glycosaminoglycans, and Anti-RAGE Antibody or Sulfated Glycosaminoglycans Delivered in Vivo Inhibit Pulmonary Metastasis of Tumor Cells. J. Biol. Chem..

[161] Billiau A., Matthys P. (2009). Interferon-γ: A historical perspective. Cytokine Growth Factor Rev..

[162] Brooks B., Briggs D.M., Eastmond N.C., Fernig D.G., Coleman J.W. (2000). Presentation of IFN-γ to Nitric Oxide-Producing Cells: A Novel Function for Mast Cells1. J. Immun..

[163] Bocian C., Urbanowitz A.-K., Owens R.T., Iozzo R.V., Götte M., Seidler D.G. (2013). Decorin Potentiates Interferon-γ Activity in a Model of Allergic Inflammation. J. Biol. Chem..

[164] Deshauer C., Morgan A.M., Ryan E.O., Handel T.M., Prestegard J.H., Wang X. (2015). Interactions of the Chemokine CCL5/RANTES with Medium-Sized Chondroitin Sulfate Ligands. Structure.

[165] Proudfoot A.E.I., Fritchley S., Borlat F., Shaw J.P., Vilbois F., Zwahlen C., Trkola A., Marchant D., Clapham P.R., Wells T.N.C. (2001). The BBXB Motif of RANTES Is the Principal Site for Heparin Binding and Controls Receptor Selectivity. J. Biol. Chem..

[166] Lee J., Rho J.-H., Roehrl M.H., Wang J.Y. (2021). Dermatan Sulfate Is a Potential Regulator of IgH via Interactions with Pre-BCR, GTF2I, and BiP ER Complex in Pre-B Lymphoblasts. Front. Immunol..

[167] Stojnić B., Galmés S., Serrano A., Sulli M., Sušak L., Seye N., Palou A., Diretto G., Bonet M.L., Ribot J. (2024). Glycosaminoglycan dermatan sulfate supplementation decreases diet-induced obesity and metabolic dysfunction in mice. BioFactors.

[168] Huang X., Lin N., Liang X., Zhang H. (2022). Dermatan sulfate and chondroitin sulfate from *Lophius litulon* alleviate the allergy sensitized by major royal jelly protein 1. Food Funct..

[169] Lafuente-Merchan M., Ruiz-Alonso S., Zabala A., Gálvez-Martín P., Marchal J.A., Vázquez-Lasa B., Gallego I., Saenz-del-Burgo L., Pedraz J.L. (2022). Chondroitin and Dermatan Sulfate Bioinks for 3D Bioprinting and Cartilage Regeneration. Macromol. Biosci..

[170] McEwan T.B.-D., De Oliveira D.M.P., Stares E.K., Hartley-Tassell L.E., Day C.J., Walker M.J., Jennings M.P., Sluyter R., Sanderson-Smith M.L. (2025). Group A Streptococcus interacts with glycosaminoglycans via M proteins to modulate bacterial adherence in vitro. FEBS J..

